# Perceived consequences of Entrustable Professional Activities implementation on the clinical skills development of Family Medicine registrars: Observations and reflections

**DOI:** 10.4102/safp.v68i1.6349

**Published:** 2026-09-08

**Authors:** Christiaan Visser, John Musonda, Daniël Joubert, Magdelena Petkova, Deidre Pretorius

**Affiliations:** 1 Department of Family Medicine, Faculty of Health Sciences, University of the Witwatersrand, Johannesburg, South Africa

**Keywords:** entrustable professional activities, EPAs, OSCE, clinical skills, training, competency-based medical education, family medicine training, assessment

## Abstract

Family medicine training in South Africa has shifted from traditional assessments to competency-based methods, including Entrustable Professional Activities (EPAs). Entrustable Professional Activities emphasise what learners can do rather than what they know, aligning with modern, more pragmatic pedagogical theories of praxis. At the University of the Witwatersrand’s Division of Family Medicine, implementation of EPAs has coincided with some potentially unintended consequences. Registrars performed poorly on Focused Objective Structured Clinical Examination (OSCE) stations during internal end-of-second-year examinations, indicating possible erosion of individual clinical skills and diagnostic precision. Using Gibbs’ Reflective Cycle, we analyse the emerging conflict among workplace-based entrustment decisions, the growing assessment burden and traditional competencies demonstrated in high-stakes examinations.

**Contribution:**

This reflective article contributes to the literature by highlighting how EPA implementation may inadvertently undermine discrete clinical skills despite satisfactory workplace-based performance and by proposing principles for recalibrating assessment. We advocate for a balanced, programmatic assessment approach to ensure the holistic development of future family physicians.

## Introduction

The move towards competency-based medical education (CBME) has transformed training for family medicine registrars.^1^ The introduction of Entrustable Professional Activities (EPAs) has led to the operationalisation of workplace-based assessment (WBAs) from the previously ill-defined state of ‘trust’ towards the more tangible observation of clinical tasks and increasing independence as registrars progress.^2^ Moreover, EPAs are intended to bridge the gap between the theoretical competence assessable in a high-stakes examination and the clinical judgement required to practice medicine safely and effectively.^3^

The University of the Witwatersrand’s Division of Family Medicine welcomed the introduction of EPAs. Our faculty members’ expectations ranged from better training outcomes to clearer judgement about when registrars are ready for independent practice. A concerning pattern, however, emerged during our recent internal examination for registrars. Registrars who had been making satisfactory progress in their EPA assessments unexpectedly performed poorly on specific, focused skills stations. This situation led us to reflect on whether our EPA implementation has unintentionally diverted attention from the development and maintenance of individual clinical competencies.

## Gibbs’ reflective cycle: A structured analysis

We used Gibbs’ Reflective Cycle^4^ to explore our experiences systematically and to make meaning of the situation at hand. Gibbs’ model was applied to critically reflect on what happened, our emotional and analytical responses, the situation’s broader implications and our potential course of action to remedy the situation.

Gibbs’ Reflective Cycle^4^ consists of six sequential yet interconnected stages, namely: Description, Feelings, Evaluation, Analysis, Conclusion and Action Plan. Gibbs prompts us to use these stages to reflect on any given experience. The model’s cyclical approach highlights ongoing improvement by enabling reflection from one stage to the next. We also selected this model as it promotes ongoing personal and professional growth.

## Description: What happened?

In 2024, the Family Medicine registrar training programme adopted a set of core EPAs that are aligned with the national family medicine curriculum. Registrars completed EPA assessments across clinical settings, such as hospital rotations and placements at community health centres. Clinical supervisors rated each registrar on how well they performed the specific professional activities. During a recent Objective Structured Clinical Examination (OSCE) cycle, we observed a notable decline in performance on stations testing focused clinical competencies. What concerned us was that these foundational clinical skills have historically been the registrars’ strengths.

Examiners reported that students performed superficial physical examination techniques, missed relevant clinical signs or cues and placed greater emphasis on context or psychosocial factors, thereby hindering systematic diagnostic reasoning. Some candidates appeared to rely more on familiarity with patient narratives than on systematic clinical decision-making. Surprisingly, many of these same registrars had received satisfactory-to-excellent EPA ratings in their workplace assessments. Their supervisors had documented progressive entrustment for activities such as ‘managing acute presentations’ and ‘conducting comprehensive patient consultations’. We realised there was a disconnect between EPA performance and OSCE outcomes. As this finding was both unexpected and concerning, it raised questions about the calibration of our assessment tools, supervisor judgement and whether EPA assessments in everyday clinical settings adequately measure diagnostic accuracy and procedural skills.

## Feelings: What were we thinking and feeling?

As faculty members and examiners, we experienced a range of emotions, moving from initial scepticism to genuine concern about our entrustment decisions. Several examiners questioned the validity of our EPA assessments when registrars entrusted with clinical duties failed to show key clinical examination skills. During informal discussions, registrars reported frustration, with many noting that their training had become a cycle of completing EPA assessments and documenting workplace activities, leaving little time for deliberate clinical examination practice. Entrustable professional activity assessments also do not have a score attached to them. Instead, supervisors assign a specific level of entrustment, based on the specific EPA.

## Evaluation

### Positive aspects

Traditional WBAs focused on individual tools (mini-clinical evaluation exercise, DOPS, logbooks and case-based discussions) linked to lists of outcomes and unit standards, which often felt fragmented and abstract to registrars and their supervisors. At the University of the Witwatersrand, the use of the Royal College of General Practitioners WBA tool^5^ since 2020 has further supported this shift by providing a structured, internationally recognised framework for describing and rating these activities. The introduction of an EPA framework has brought several benefits to our registrar training programme. Entrustable professional activities re-organise assessment around clearly described units of work (for example, managing a patient with undifferentiated acute illness in primary care) that integrate knowledge, skills and professional behaviours in authentic, real-life clinical practice. The EPA framework also provides an easily accessible online format with real-time access and a shared language for supervisors and registrars to discuss what progressive independence might look like in different clinical contexts.

The electronic portfolio of learning ensures real-time and comprehensive assessment. Late submissions of work plans and continuous assessments cannot be manipulated in the electronic format, and any unreasonable delays in registrars’ academic outputs are therefore readily identifiable. Entrustable professional activities appear to have improved our ability to notice when a registrar is struggling to keep pace with the group. Because each professional activity is clearly articulated, gaps in a registrar’s performance become more visible, which allows us to provide more precise remediation pathways. Yet, by specifying minimum observable standards for entrustment, the framework risks narrowing attention to discrete tasks, rather than the broader, more nuanced judgement, adaptability to work independently and reflective thinking that also characterise a competent family physician.^6^ This concern was echoed in registrars’ own narrative reflections, in which several described their training as a continual cycle of completing EPA forms and documenting activities, with limited opportunity for deliberate practice of core examination skills or deeper clinical reasoning.

Entrustment decisions, especially summative EPA entrustment, rely on higher-level inferences and thus require more than direct observation or checklist data. Gut feelings and pattern recognition, grounded in supervisory experience and contextual knowledge, are often considered to be legitimate components of entrustment, especially in ad hoc decisions, provided they are made visible and reflected upon.^7^

### Negative aspects

When assessment systems emphasise holistic workplace tasks, there may be a risk that discrete logbook skills could be neglected.^8,9^ Our observation of the apparent loss of individual clinical skills serves more as a red flag than as definitive evidence of harm, prompting concern that some individual clinical skills might be receiving less focused attention within our current EPA implementation. Highly holistic workplace assessments may, especially under time and service pressures, lead supervisors to complete EPA forms as general checklists of overall performance. In these circumstances, attention to specific technical competencies can be diluted, and opportunities for targeted, competency-focused feedback and learning are reduced.^6^ In our setting, this emerging signal has heightened awareness of the need to intentionally safeguard logbook skills within an EPA-based framework. The requirement for registrars to gather multiple EPA assessments across various settings may lead to a ‘tick-box’ mentality, where the emphasis may inadvertently shift from gaining a deep understanding to a purely administrative exercise.^6^ Although feedback is intended to be central to EPA-based assessment, in our context we have sometimes felt pressured to prioritise the completion of EPA forms over engaging in rich feedback and teaching conversations. In this opinion piece, we reflect on these experiences and the risk that administrative and service demands can inadvertently shift attention away from the formative purpose of EPAs towards form completion. Registrars who develop a constant sense of judgement may find their opportunities for low-stakes practice and skill-building severely limited, ultimately hindering their professional growth and personal well-being.^10,11^ Feedback is, however, not intended to elicit judgement, but human nature may perceive negative feedback as judgement.

The disconnect between EPA ratings and focused OSCE performance in this cohort prompted us to question how robust our workplace-based entrustment decisions are and to consider potential contributing factors such as registrar cohort characteristics, learning platform differences and supervisor training, rather than assuming either workplace-based assessment or OSCE performance to be inherently more valid.^12^

If supervisors rated registrars highly on EPAs that implicitly required strong logbook skills, yet these skills were demonstrably weak, this pattern may reflect influences such as social desirability bias and a tendency to avoid negative documentation, thereby challenging the extent to which EPA assessments captured the clinical competence they purported to measure.^11^ This may have major implications if we want to move away from summative assessments towards entrustable activities as a criterion for promotion.

## Analysis: What sense can we make of the situation?

Several factors could explain the disconnect between the EPA performance and OSCE outcomes we observed. Figure 1 depicts our conceptual framework, demonstrating the disconnect we perceived between EPA performance and OSCE outcomes. The central gap between the two is widened by the following five factors that we identified: Fragmentation of holistic competence, assessor readiness and competence for EPAs, a shift in learning focus, limitations of WBAs and the differing nature of the OSCE construct.

**FIGURE 1:**
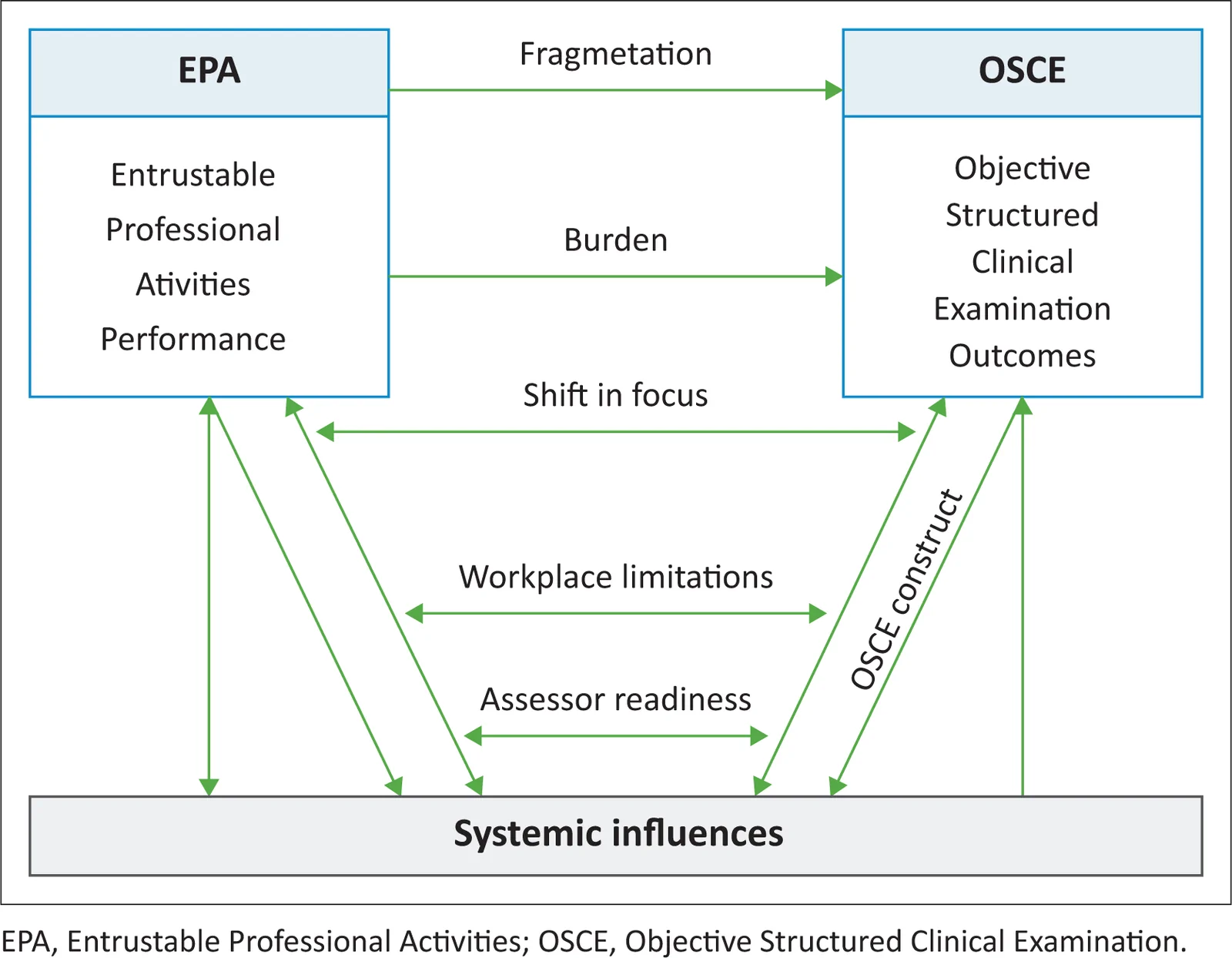
Conceptual framework of how Entrustable Professional Activities implementation may contribute to a disconnect between workplace entrustment decisions and Objective Structured Clinical Examination-measured clinical skills in family medicine registrar training.

### Fragmentation of holistic competence

In family medicine, a comprehensive patient encounter requires the use of multiple integrated clinical competencies. These skills include taking patient histories, conducting physical examinations, making a diagnosis, managing patient care, communicating effectively and demonstrating professionalism. Within EPAs, the observable practice activities (OPAs) are discrete, while the EPA as a whole includes the holistic components.^13^ Discrete activities may at times not receive the level of attention they deserve. The current assessment strategy provides a much more detailed and useful clinical dataset. However, in our early implementation, some registrars and supervisors reported that the way EPAs were operationalised and documented made them feel more like checklist exercises than holistic judgements of readiness for practice, reflecting partial misunderstanding of EPA principles and the need for ongoing faculty and registrar development in our setting.

While the granularity of EPAs is valuable for workplace observation, it may also fragment the holistic view of clinical competence.^10^ When an assessment emphasises the overall activity (for example, ‘managing an acute presentation’), individual components (such as ‘performing a comprehensive cardiovascular examination’) may be assumed rather than explicitly observed and assessed. Each EPA is explicitly linked to a large number of underlying logbook skills. In our national context, more than 400 discrete skills are aligned with 22 EPAs. In principle, this design recognises that it would be impossible to assess all of these skills comprehensively in a single OSCE, and that iterative workplace-based assessment over 3 years offers a more feasible (though still imperfect) way to sample and develop them. However, our experience suggests a potential implementation gap: Some supervisors and registrars appeared to focus on documenting EPA completion while implicitly assuming that the associated logbook skills had been mastered, rather than consistently observing and giving feedback on those skills. This may partly explain the disconnect we observed between satisfactory EPA ratings and weaker performance on focused skills stations and highlights the importance of ensuring that both supervisors and registrars understand and actively work with the specific skills connected to each EPA.

### Assessor readiness and competence for entrustable professional activities

We also need to consider how often assessments occur and the administrative tasks or paperwork they create. The increased frequency of EPA assessments may have led to a change in strategic behaviour among both registrars and supervisors. When assessment becomes burdensome, supervisors and registrars may resort to ‘workarounds’ and prioritise completing evaluations over maintaining core skills. Our observations are consistent with international reports of tick-box behaviours and a narrowing of competence when EPAs are implemented without sufficient attention to foundational skill assessments.^6,14^ Because supervisors often lack time, they may rate registrars based on general impressions instead of directly observing these skills.

### Shift in learning focus

Entrustable professional activities are meant to balance trust and independence in real clinical tasks. However, their focus may have subtly changed how registrars think about their own learning. Instead of refining basic examination skills, some may be drawn more to completing assigned activities and demonstrating autonomy. For example, in our training platform, registrars and supervisors have reported that when there are few targeted assessments of specific examination systems, such as the cardiovascular examination, these skills tend to receive less deliberate, focused practice and observation. Through our regular engagement with registrars in teaching and supervisory settings, we have encountered concerns that foundational skills may be perceived as ‘already covered’ within the broader umbrella of EPA work, potentially reducing the explicit attention given to these fundamentals.^15^

### Limitations of workplace-based assessments

While they are authentic, WBAs are inherently variable and context dependent.^16^ In our demanding clinical settings, performing comprehensive physical examinations may be condensed or even omitted in favour of focused assessments guided by a patient’s clinical context. While focusing examinations on the presenting problem is appropriate in demanding clinical settings, registrars in our programme have perceived that service pressures can make it harder to find protected moments for full, systematic examinations that are closely observed and debriefed, and we therefore raise this as a local concern rather than a generalisable claim.

Although EPAs in our national framework are designed to integrate patient-centred communication, examination and technical skills, our local experience suggests that, in practice, the global management aspects sometimes received more attention than the underlying discrete skills, which were occasionally assumed rather than explicitly observed and coached.

### The Objective Structured Clinical Examination as a different construct

Objective Structured Clinical Examinations assess performance under standardised, controlled conditions and require students to demonstrate their skills on demand without the contextual cues of real-life clinical practice.^17^ It is possible that competent registrars in workplace settings may struggle to transfer their skills to the artificial OSCE environment. While assessment anxiety undoubtedly contributes to performance in high-stakes OSCEs, our experience as examiners in this cohort was that the pattern of underperformance appeared more substantial than we would usually attribute to anxiety alone. This local observation led us to consider the possibility of gaps in some Master of Medicine foundational clinical skills (for example, core logbook skills in history-taking, physical examination and basic procedures that form the backbone of the family medicine curriculum), rather than to claim definitive proof of such deficits.

## What else could we have done?

Several alternative approaches to facilitation and assessment emerged during our reflective process. We reflected on whether it might make more sense to assess our registrars directly on physical exam techniques, rather than leaving them implied. Striking a balance between what registrars do in real life (during EPA evaluations) and what they demonstrate in a structured setting (like an OSCE) seemed appropriate. We also deliberated on various strategies to improve our supervisor training.

Dedicating specific opportunities for registrars to practise hands-on skills or to participate in structured, formative mock OSCEs that do not carry summative consequences may provide a more realistic way to improve their focused techniques than relying solely on opportunistic encounters in routine service. We also reflected on how to track registrars’ progress over time. Within the national framework, 360-degree assessments provide rich information on professionalism, and the electronic Dashboard in the portfolio aggregates registrar and supervisor feedback, which should be interrogated at regular intervals (approximately every 5 months) by the local Clinical Competence Committee (CCC) to identify early warning signals. In our context, however, we were concerned that if these data are engaged with mainly as a record-keeping requirement rather than interrogated in depth, subtle gaps in discrete clinical skills may still be missed, despite the presence of these structures. None of these ideas is a quick fix. Any of them could easily fail without careful rollout and team support. Still, these were among the options the team considered as we tried to understand the gaps we identified.

## Action plan: What will we do differently?

We will implement the following changes in our programme:

### Implement a programmatic assessment framework

Blend workplace-based EPAs, formative OSCEs, summative exams, portfolio reviews and competence-committee deliberations to give a more rounded view of registrars’ competence.^18,19^

### Reinstate regular skills assessments throughout training

Bring back regular, formative assessments of clinical skills throughout training. This may include quarterly mini-OSCEs, direct observation of procedural skills (DOPS) and structured clinical examinations during teaching sessions. These assessments will not replace EPAs but will complement them by ensuring that essential skills remain a priority.

### Revise entrustable professional activity descriptors

Rather than adding more checklist items, we will refine the existing EPA descriptors so that they briefly but explicitly describe the expected standards of physical examination and clinical reasoning at each level of entrustment. Supervisors and assessors will be encouraged to use and periodically revise these descriptors and to familiarise themselves with the required standards and level of entrustability for physical examinations and diagnostic reasoning, so that their global judgements and narrative feedback consistently address these core competencies.

### Reduce assessment burden

Streamline our EPA assessment requirements, focusing on quality over quantity. Rather than requiring registrars to accumulate numerous EPA assessments, we will emphasise meaningful observations and rich narrative feedback. Reducing administrative tasks will create time for deliberate practice. The current national expectation is 1–2 WBAs per workweek. Within that 1–2 h, the focus should be on quality, reflection and feedback.

### Faculty development

Retrain supervisors to ensure comprehensive feedback and a good standard of practice across the extended training platform.

### Protected learning spaces

Registrars must navigate a complex service-delivery setting that also serves as training. Protected learning spaces are therefore essential to master focused skills. We will set aside time for regular skills laboratory sessions, peer teaching opportunities and formative practice or ‘mock’ OSCEs. These spaces will be designated as low-stakes learning opportunities, separate from formal assessments. In our context, *low-stakes* refer to formative assessment activities that prioritise feedback and reflection, with little or no direct impact on progression or grading. These can include ad-hoc WBA encounters when used mainly for coaching. By contrast, *formal assessments* are planned, documented assessments (including specified WBAs) that contribute to summative decisions such as entrustment, progression or certification.

### Monitor and evaluate

Implement regular, CCC-led monitoring of assessment outcomes, in which annual OSCE performance trends are considered alongside EPA ratings, portfolio and 360-degree data and qualitative operational feedback from registrars and supervisors, with all these sources subject to ongoing validation and calibration within a programmatic assessment approach. In our setting, this monitoring will be supported by ongoing participatory action research to standardise assessment: Supervisors, registrars and CCC members engage in repeated cycles of joint case-based calibration meetings, review of real assessment data and refinement of EPA descriptors and logbook expectations, with faculty-development activities updated in response to what is learned.

At present, we do not know to what extent our current EPA ratings capture the same aspects of clinical competence as our focused OSCE stations. In this context, we use ‘clinical competence’ to refer to the specific constellation of abilities emphasised by each method.^12,13^ For example, OSCEs emphasise standardised demonstration of discrete history-taking, examination and reasoning steps under controlled conditions, whereas EPA ratings reflect supervisors’ global judgements of performance in real clinical encounters over time. Ongoing evaluation of how these different data sources relate to one another in our programme will be important to detect unintended consequences early and to adjust our assessment approach accordingly.

### Engage in scholarly dialogue

Share our experiences and findings with the South African family medicine community. We want others to join this conversation about EPA implementation and about how to achieve a balanced assessment system in postgraduate training that combines structured assessment of core clinical skills (for example, OSCEs and direct observation of procedures) with holistic, workplace-based EPA judgements of integrated, patient-centred care. In addition to sharing insights, there is a need for empirical research that examines the impact of EPA implementation on discrete clinical skills, OSCE performance and programmatic assessment outcomes in South African family medicine training. Through such collaborative research and dialogue, the family medicine community can move beyond anecdotal concern to evidence-informed refinement of EPAs in postgraduate training.

## Discussion

Our experience is unlikely to be unique. As South African family medicine training transitions to EPAs and CBME frameworks, we need to remain alert to unintended consequences such as fragmentation of competence, assessment burden and behaviours that do not support learning.^6,8,9,10,14^ Entrustable professional activities capture what happens in real-world clinical settings effectively and help decide when a registrar is ready to assume further responsibilities. While the relevance of EPAs in our postgraduate teaching is clear, we must also acknowledge their limitations.

There is an ongoing debate about whether the discrete building blocks of clinical expertise may be overlooked when we rely too heavily on workplace-based evaluations.^6,8^ Family physicians must juggle both the art of delivering a seamless, patient-centred consultation and proficiency in technical skills. We therefore advocate for a balanced assessment approach that supports both the workplace authenticity of EPAs and the rigour of structured clinical examinations. This balance is not a compromise but a recognition that different assessment methods serve various purposes and capture different facets of competence.^18,19^

Programmatic assessment, which integrates multiple data sources and emphasises longitudinal decision-making by competence committees, offers a promising framework for achieving this balance.^20^ The assessment burden accompanying CBME implementation warrants attention. More assessments may not necessarily yield better outcomes. Quality and meaningful feedback should guide our assessment design, not the quantity of data points collected. In our context, streamlining low-value, duplicative documentation (including some e-portfolio and service-related administrative tasks) would not reduce the expected 1–2 h of WBA and portfolio work but would repurpose that time towards higher-value activities such as direct observation, feedback and deliberate practice. The success of any assessment system depends on the observations, feedback and judgement provided by supervisors. Faculty need capacitation and time to fulfil these roles effectively. Without this investment, even well-designed assessment frameworks will fail to achieve their intended outcomes.

## Conclusion

Our subjective experience pre-empts unintended consequences when assessment reform is implemented without specific attention to balance and integration. The apparent decline in some individual clinical skills in this cohort has functioned as a local cautionary signal for our programme, prompting us to reflect critically on how we implement EPAs and structure assessment. We do not advocate for abandoning EPAs, but for using them as part of an integrated assessment approach that provides high-quality feedback, creates deliberate learning opportunities and safeguards the development of core clinical competencies. Assessment should serve learning, not the other way around. A balanced approach that combines authentic workplace experience with structured skills assessment may better support the development of registrars’ clinical competence across different domains.
